# Editorial: Up to date developments of nanocellulose towards materials with medical benefits

**DOI:** 10.3389/fbioe.2022.1041826

**Published:** 2022-09-27

**Authors:** Selestina Gorgieva, Diana Ciolacu, Stanislaw Bielecki

**Affiliations:** ^1^ Institute of Engineering Materials and Design, Faculty of Mechanical Engineering, University of Maribor, Maribor, Slovenia; ^2^ Department of Natural Polymers, Bioactive and Biocompatible Materials, Petru Poni” Institute of Macromolecular Chemistry, Iasi, Romania; ^3^ Institute of Molecular and Industrial Biotechnology, Faculty of Biotechnology and Food Sciences, Lodz University of Technology, Lodz, Poland

**Keywords:** nanocellulose, bacterial cellulose, plant nanocellulose, wound dressing and biomedical applications, medicine

**FIGURE 1:**
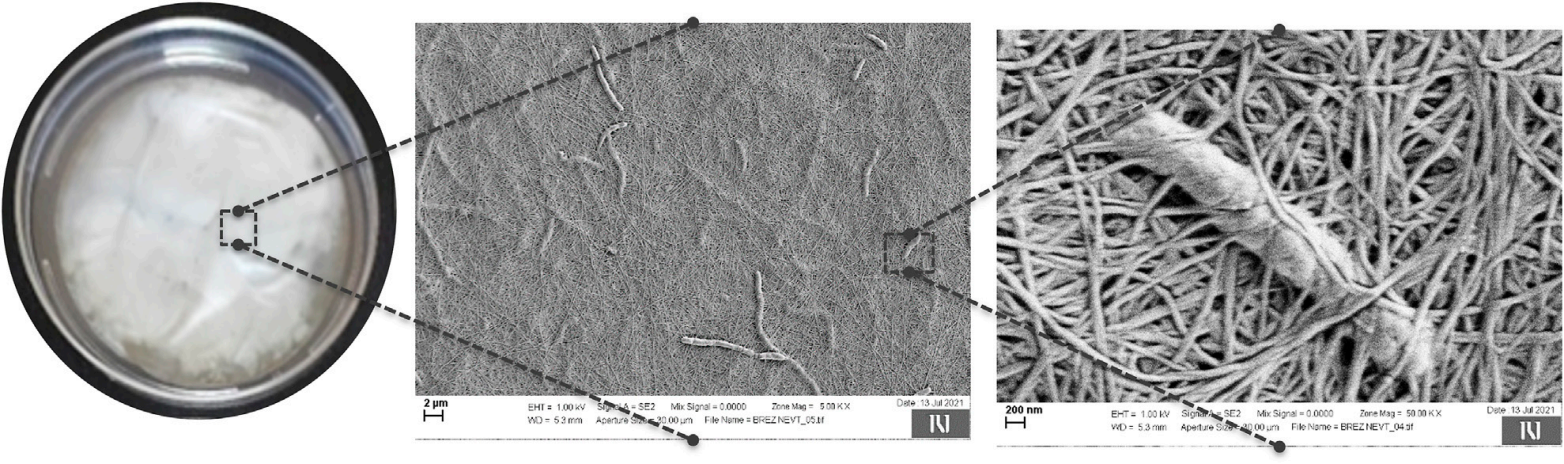
Bacterial cellulose network with entrapped cellulose producing bacteria.

In simple terms, nanocellulose is a nano-scaled, ordered form of cellulose obtained either by bottom-up, biotechnological processes (bacterial cellulose, Figure 1) or more destructive, top-down (plants, trees … ), chemo-mechanical approaches. As such, it delivers a distinctive combination of appealing properties, such as high surface area, hydrophilicity, mechanical strength, abundant hydroxyl groups, porosity, high crystallinity, purity, and in particular cases (bacterial cellulose) biocompatibility, altogether making it a highly unique material, fitting to diverse disciplines (Gorgieva, 2020, Gorgieva and Trček, 2019). The latest knowledge related to the nanoform of the most abundant natural polymer defines it as a material of the future!

The collection of articles under the Research Topic: *Up to Date Developments of Nanocellulose toward Materials with Medical Benefits* features reviews and original research articles, discussing the state of the art, the challenges, impediments, and applicability in various forms, for medical purposes. It aims to uncover the recent advances in nanocellulose-based materials development and their use in specific and demanding medical applications. The Research Topic comprehensively discussed the limitations of nanocellulose in biocomposites (Masek and Kosmalaska), the current position of nanocellulose within the cartilage tissue engineering discipline (Szustak and Gendaszewska-Darmach), its perspective within the exciting field of neurological disorders treatment (Johra et al.), reconstructive surgery and wound healing (Jankau et al.). Space is given also to discovery of new type of nanocellulose-composite out of moso bamboo and dopamine, developed for packaging applications (Wu et al.).

In the publication “*Technological limitations in obtaining and using cellulose biocomposites*” by Masek and Kosmalska, several essential data, from specific properties to applications, are gathered in one place, providing a basis for comparing different (bacterial or plant) types nanocellulose. Bacterial cellulose has a unique advantage over nanocelluloses obtained from plants and, in addition, a higher economic value, as well as lower environmental impact. Being a biomaterial with high chemical reactivity and with special magnetic and electrical properties, bacterial cellulose is considered one of the materials of the future in the medical industry (Nicu et al., 2021). On the other hand, the review article aims to present a more comprehensive perspective that can help decide which type of cellulose may be more suitable for a particular purpose.

Szustak and Gendaszewska-Darmach offer a comprehensive synthesis of research on nanocellulose-based biomaterials used to construct scaffolds for cartilage tissue engineering in the review article “*Nanocellulose-Based Scaffolds for Chondrogenic Differentiation and Expansion*”. Repairing cartilage tissue remains a severe clinical challenge because of its limited regeneration capacity resulting from lack of vascularization, lymphangion, innervations, and restricted infiltration of local progenitor cells. Moreover, the cartilage is sparsely populated with chondrocytes embedded within an extracellular matrix. Since nanocellulose scaffolds have been shown to induce the differentiation of stem cells into chondrocyte phenotypes, this crucial feature makes it an excellent scaffolding material. The review discusses the current state of knowledge regarding nanocellulose and its composites as scaffolds for chondrogenic expansion and differentiation. The promotion of cellular interactions and the development of tissues that mimic the extracellular matrix, imply on bacterial cellulose potential in cartilage tissue engineering, even the field is mainly at the laboratory stage. As such, can potentially improve the quality of life and comfort and can be considered part of a new generation of nanomaterials for biomedical applications.

The challenge for chemists and biotechnologists to produce a cellulosic material to meet the versatile needs of soft tissue reconstruction, and be used as an insert in various forms in the human body is presented in the article “*Bacterial Cellulose Properties Fulfilling Requirements for a Biomaterial of Choice in Reconstructive Surgery and Wound Healing*”. Experimental results consistent with previous reports using an acellular dermal matrix are presented, confirming that nanocelluse does not cause adverse effects, such as ulceration or extrusion. The review also summarizes the characteristics of cellulose produced by different strains of the *Komagataeibacter* genus and the characteristics of bacterial cellulose and its composites used in wound care, which were obtained from clinical studies. The additional research activities are needed to translate current knowledge from *in vitro* and *in vivo* laboratory research studies to human trials and implicitly to the commercialization of materials that could replace missing soft tissues.

The potential of biomaterials for treating neurological disorders is described in the article “*Perspective Insights to Bio-Nanomaterials for the Treatment of Neurological Disorders*”. Much attention is being paid to hydrogels as potential candidates for treating common neurological disorders, such as Alzheimer’s disease, Parkinson’s disease, spinal cord injury, stroke. Hydrogels offer advantages such as flexibility and porosity and mimic the properties of the extracellular matrix of the central nervous system. These factors make them an ideal scaffold for drug delivery across the blood-brain barrier and tissue regeneration using stem cells. There are limitations to using nanomaterials, such as the varied solubility at different temperatures, the toxicity of synthetic materials, the possible diffusion of biomaterials in non-specific neural areas and most importantly, the high cost. However, it is essential to note that biomaterials are the most practical alternatives to the existing treatment strategies available for neurological disorders and that the choice of combined therapies, unique in a given medical scenario, is the solution to this issue.

Bacterial cellulose is highly hydrophilic, due to propensity of hydroxyl groups, yet some applications (as packaging) strictly required reduced water attraction/holding capacity in order to protect the product being packed (food, drug, medical device.). The last article in this series, “*A Superhydrophobic Moso Bamboo Cellulose Nano-Fibril Film Modified by Dopamine Hydrochloride*”, describes how bamboo powder was used as a substrate and a composite film of cellulose nanofibers was chemically modified with dopamine to prepare a film with excellent superhydrophobic and self-cleaning properties. It was used as a functional material to preserve the natural characteristics of bamboo powder. This study theoretically discussed an environmentally friendly type of protection, suggesting using the final composite in the packaging industry.
